# Albert C. Broders, tumor grading, and the origin of the long road to personalized cancer care

**DOI:** 10.1002/cam4.3112

**Published:** 2020-05-07

**Authors:** James R. Wright

**Affiliations:** ^1^ Departments of Pathology & Laboratory Medicine and Paediatrics University of Calgary Alberta Children's Hospital Calgary Alberta Canada

**Keywords:** Albert C. Broders, David P. von Hansemann, Louis B. Wilson, medical history, precision cancer care, tumor grading and staging

## Abstract

The roots of precision cancer therapy began at the Mayo Clinic in 1914 when surgical pathologist Albert C. Broders began collecting data showing that cancers of the same histologic type behaved differently. In March 1920, based upon 6 years of clinical follow‐up, Broders published his first paper, utilizing data from over 500 cases of squamous cell carcinoma of the lip that he had blindly divided into four histologic grades based upon degree of differentiation, showing that numerical tumor "grading" allowed him to predict patient prognosis. Before this, surgeons had no scientific way to evaluate prognosis. Broders then replicated his work using other types of tumors at other body sites, as did several Mayo Fellows and pathologists at other institutions. Cuthbert Dukes in London, England not only replicated Broders’ findings with rectal adenocarcinomas, he also used the same data to develop the first tumor “staging” methodology by focusing upon depth of local invasion and presence or absence of lymph node metastases. Soon, tumor grading, tumor staging, or the combination of both represented state‐of‐the‐art prognostic techniques for scientific cancer care. This brief historical vignette celebrates the 100th anniversary of Broders’ first paper, which is the starting point for the long road to personalized cancer care.

## Graphical abstract

The roots of precision cancer therapy began 100 years ago when surgical pathologist Albert C. Broders began collecting data showing for the first time that cancers of the same histologic type and anatomical site behaved differently. He had blindly divided squamous cell carcinoma of the lip into four histologic grades based upon degree of differentiation, showing that numerical tumor "grading" allowed him to predict patient prognosis. This quickly led to "staging" of cancers.

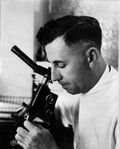

This brief historical commentary celebrates an event that has previously been described by some cancer historians,^1^, ^2^ but provides some additional context making it worthy of special recognition because of its upcoming 100th anniversary. An article published in the *Journal of the American Medical Association* (*JAMA*) in 1920^3^ represents the starting point for the long road to personalized cancer care. This journey began with the realization that cancers, even of the same site and histologic type, behave differently and that pathological testing based upon sampling the patient's tumor could help predict the behavior of his/her cancer. After 1920, the behavior of cancers and patient prognosis could increasingly be predicted using histology; this was a huge advance in cancer patient care!

The roots of precision cancer therapy began at the Mayo Clinic in 1914 when surgical pathologist Albert C. Broders (1885‐1964) (Figure 1) began collecting data showing that cancers of the same histologic type behaved differently; in March 1920, based upon 6 years of clinical follow‐up, Broders published his first paper, utilizing data from 537 cases of squamous cell carcinoma of the lip that he had blindly divided into four histologic grades based upon degree of differentiation, showing that numerical tumor "grading" allowed him to predict patient prognosis.^1^, ^3^ He quickly replicated this study with other long‐term studies showing the predictive value of numerical grading of tumors in patients with squamous cell carcinomas of the skin, carcinomas of the genitourinary organs, and head & neck carcinomas.^4^, ^5^, ^6^ Broders' work was quickly followed by a flurry of other studies on this topic which have been reviewed elsewhere.^1^ One of these was by Cuthbert Dukes at St. Mark's Hospital in London. In the early 1930s, Dukes was able to replicate Broder's findings with numerical tumor grading of rectal adenocarcinomas.^7^, ^8^ He followed over 500 patients for at least 3‐5 years after resection, and like Broders, demonstrated that patients with low grade rectal adenocarcinomas fared much better than patients with high grade rectal adenocarcinomas. However Dukes also saw an alternative interpretation for the same data, noting that the depth of tumor invasion (ie, into vs. through the bowel wall) and extent of spread (ie, presence or absence of lymph node metastases) at the time of surgery was an even better predictor of rectal carcinoma patient survival than was grading.^7^

**Figure 1:**
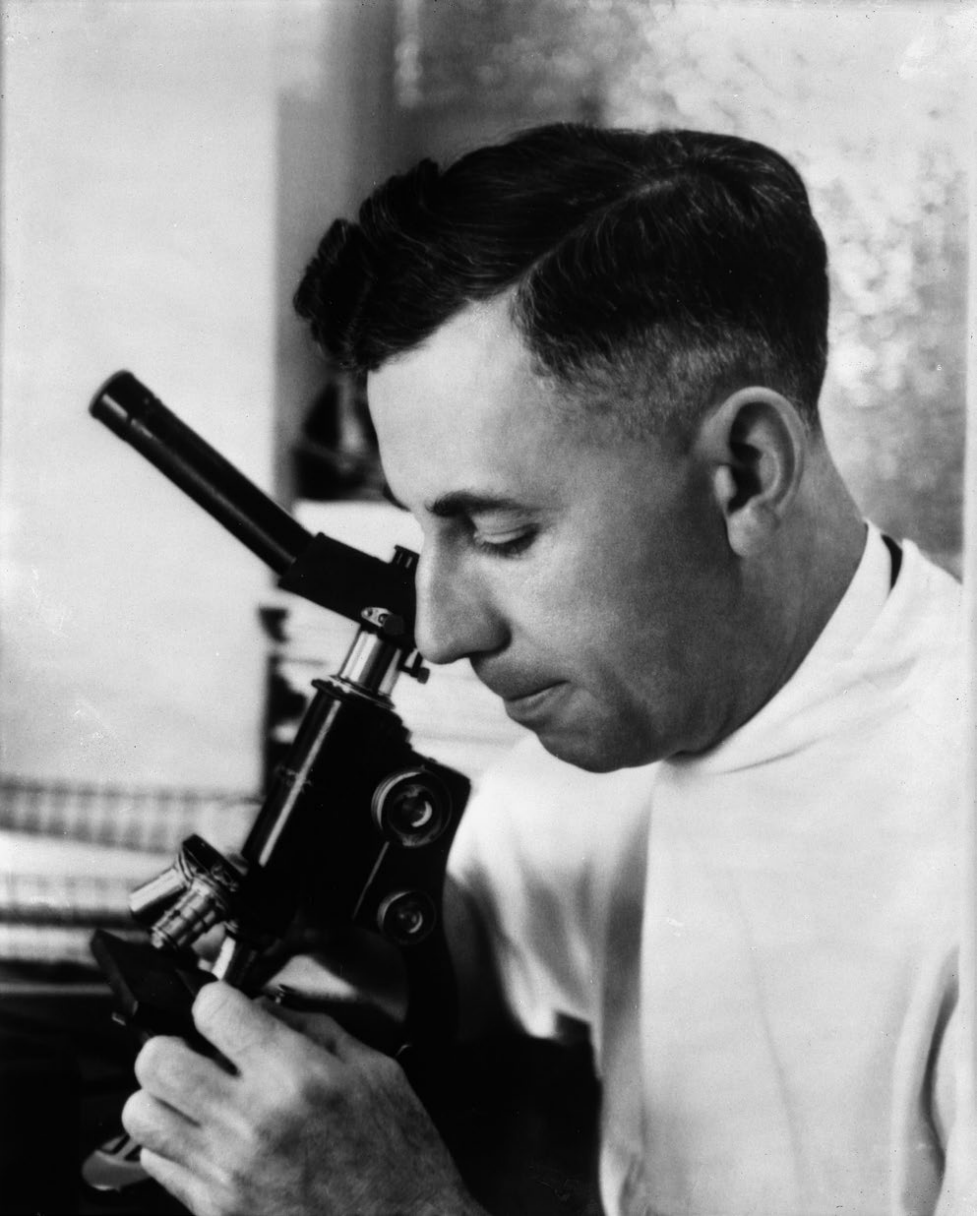
Albert Compton Broders. Credit: Mayo Historical Unit

As Dukes explained when reviewing his data in a later paper:

At first sight these figures would seem to show that the less differentiated types of malignant growths, such as Grades 3 and 4, grow more rapidly and kill the patient more quickly than the better differentiated tumours of Grades 1 and 2, but before accepting this seemingly obvious conclusion, allowance must be made for the extent of spread at the time of operation. We are not watching the natural uninterrupted march of malignant disease. These patients all underwent the operation of excision of the rectum for cancer and in some cases the disease was at an early stage and in others was actually beyond the scope of surgery at the time of operation. Looked at from this point of view, it might be argued that the survivors in each grade simply represent the proportion of cases which were at a curable stage at the time of operation. In other words, these striking differences in the numbers of deaths in each grade may not be a reflection of the rate of growth of the tumours but simply a measure of the proportion of patients in each grade who could be cured by the treatment they received.^8^

Broders, who had previously published on the utility of grading rectal carcinomas,^9^ published a follow‐up study in 1940 demonstrating that grading and “staging” were complimentary techniques for scientific prognostication, and this paper is now considered a classic article in colonic and rectal surgery.^10^ Nonetheless, standard reference books on the history of cancer have rarely even mentioned the importance of grading. For instance, Siddhartha Mukherjee, in the *Emperor of All Maladies: A Biography of Cancer*, briefly mentions staging in a general sense but not grading,^11^ the first step on the pathway to personalized cancer care.

It should be noted that it was important that Broders worked in a high profile and supportive environment that was favorable for knowledge translation. Many hundreds of surgical fellows, pathology fellows, and visiting surgeons would have been exposed to his ideas during their time training at or visiting the Mayo Clinic. When they left to return home or to take on new positions, they transplanted Mayo innovations such as intraoperative frozen section diagnosis and tumor grading.^1^, ^12^ Furthermore, the Mayo Clinic was very proactive about promoting its discoveries and innovations.^13^

While there were some credible critics opposed to grading malignancies,^14^ by the late 1930s, tumor grading and staging were both generally considered state‐of‐the‐art prognostic techniques for scientific cancer care.^1^ Nevertheless, tumor grading was a totally radical idea, as up until this time, cancer had been strictly defined by behavior (ie, the ability to invade and metastasize) and not by tumor morphology. In fact, throughout his entire lifetime, the father of cellular pathology Rudolf Virchow (1821‐1902), as well as most other prominent German pathology professors, did not even believe cancer cells were cytologically different than normal cells (ie, they believed that only their behaviors differed); pathologists who said otherwise were considered heretics.^15^ Recognition that cancer cells were morphologically different than normal cells became more mainstream beginning in the late 1920s, launching the field of cytopathology.^15^ Broders also helped precipitate one other paradigm shift, changing the very definition of cancer when he published a paper in *JAMA* in 1932 describing carcinoma in situ.^16^ After this, cancer could no longer be defined simply by invasion and metastases.

Today there are hundreds of grading and staging schemes for various types of cancer. The whole cancer biomarker field progressively evolved from Broders’ demonstration that histopathology could provide prognostic information relevant to the treatment of individual patients and that not all patients with the same histologic type, grade, and stage of cancers respond in the same way to the same treatments. In 1940, Broders demonstrated the complimentary relationship of grade and stage for rectal cancers. With the publication of the current 8th edition of the *American Joint Committee on Cancer (AJCC) Cancer Staging Manual*, AJCC staging now uses biologic information (including grade) in addition to Tumor, Node, Metastasis (T, N, M) staging for six types of cancer, as this has been shown to improve prognostication.^17^ Now, in many instances, molecular testing can help further stratify patients for optimal cancer therapies.

It is also important to recognize that the timing of Broders' work was absolutely critical for its rapid acceptance and wide‐spread implementation, as the early 1920s was a time period in which hospital‐based clinical pathologists were unified in a fight for their profession's very survival.^18^ In the decade before the United States entered World War I (WWI), most laboratory services available to American physicians and surgeons were provided by private "mail order" companies specializing in clinical pathology testing, and not by hospital‐based pathologists. Laboratory tests were considered a "commodity" that could be provided by unregulated commercial laboratories, and a city like Chicago had at least eight of these.^18^ In most hospitals, it was normal for surgical specimens, including cancers, to be discarded after surgery.^12^, ^18^ Examination of tissues by a pathologist was distinctly unusual but, if specially requested by the surgeon, histopathologic diagnostic services were provided by state public health laboratories; such services, which were free but very slow, existed in more than half of the states.^19^ The Mayo Clinic had been ahead of its time by hiring their first full‐time pathologist, Louis B. Wilson (1866‐1943),^20^ in 1905 and it quickly had a state‐of‐the‐art clinical laboratory service.

The United States entered WWI in April 1917; prior to this time, most American physicians and surgeons had never directly interacted with a pathologist and had little understanding of the scope of services pathologists could provide. However, Wilson, the founding director of laboratories at the Mayo Clinic, organized and made operational the laboratory services for the American Expeditionary Forces (AEF). In Europe, Wilson developed over 300 highly efficient pathology laboratories at AEF hospitals scattered around France which provided personalized laboratory services, directly addressing patient care issues.^21^ Laboratory medicine, which had never played a significant role in any previous war, became so critical for maintaining troop "war readiness" that all warring factions, on both sides, developed truck‐based mobile laboratories that could be driven to and from the front.^21^, ^22^ During America's short involvement in WWI, AEF physicians and surgeons developed increased expectations related to both the quality and availability of pathology and laboratory services and they brought these expectations home with them when they returned to the United States.^18^, ^21^ This was fortunate for the American College of Surgeons (ACS), which had formed in 1912 with the goals of uplifting surgical practice and standardizing hospitals.^23^, ^24^ After the War, the President of the United States awarded Wilson (and also his commanding officer) Distinguished Service Awards; Wilson's citation read in part “by reason of his exceptional organizing and executive ability he organized most efficiently pathological service throughout the AEF that was of inestimable value to the medical and surgical services.”^21^ The ACS immediately invoked Wilson's services to help develop minimum standards for hospital‐based laboratory services.^24^

In 1922, hospital‐based pathologists organized and formed the American Society of Clinical Pathologists (ASCP); their membership was looking to find value‐added services that they could provide, such as intraoperative frozen section diagnosis, surgical pathology, and autopsies, that could not be readily provided by their competitor, mail order commercial laboratories.^18^ The leadership of ACS and the ASCP developed a symbiotic relationship that rapidly transformed hospitals and their laboratory services.^18^, ^24^ Changes in laboratory medicine practice in the roaring twenties facilitated the rapid spread of cancer diagnostic innovations like intraoperative frozen section diagnosis and tumor grading as these kinds of breakthroughs helped promote and justify a switch from a private commercial laboratory model to a hospital‐based laboratory model, which could provide consultations and more personalized services. After 1927, the ACS hospital standardization process mandated pathological examination of all surgical specimens, essentially creating the new subspecialty of surgical pathology.^12^, ^18^ While it was not mandatory for the US hospitals to meet ACS standards, there was huge pressure, both reputational and financial, as surgeons who were ACS Fellows would normally not operate in hospitals that were not on the ACS approved list. In fact, it took only a few years for over 90% of large (>100 beds) United States and Canadian hospitals to become ACS approved.^24^

Before ending this historical vignette, it is important to digress and briefly discuss David Paul von Hansemann (1858‐1920) (Figure 2), his theory of anaplasia, and its potential application. Hansemann, who had trained with Julius Cohnheim (1839‐1884) and Virchow, was a professor of pathological anatomy at the University of Berlin in 1890, when he described his concept of anaplasia,^25^ He believed cancer cells were characterized by loss of differentiation and asymmetrical mitoses, and that these features could be assessed histologically.^26^, ^27^, ^28^ His ideas were highly disputed by almost all German academic pathologists, who, as mentioned earlier, did not believe in the uniqueness of cancer cells but rather were focused on their behavior (ie, metastases and invasion). While not the first to describe the histologic features of malignant cells,^15^ Hansemann, armed with his novel concept of anaplasia, went on to describe these features in a much clearer manner than before. While Hansemann equated anaplasia with malignancy and noted that some cancers were more anaplastic than others,^25^, ^29^ he did not progress beyond this and develop the highly practical concept of grading, likely because his “footing” was too tenuous to take this next step. Had he been working in a supportive environment, rather than battling with Virchow and his followers, likely the first step on the long road to personalized cancer care would have been taken by him. Instead, Hansemann's ideas were soon forgotten,^25^, ^30^, ^31^ and so had no bearing on what happened next. Only recently has it been recognized that many of his ideas were actually correct and those of his detractors were not.^25^ While Hansemann was first to describe the concept of anaplasia that was fundamental to Broders’ grading hypothesis, Broders had needed to “rediscover” anaplasia on his own over two decades later.^1^ Only after publishing his tumor grading papers did Broders learn of Hansemann from James Ewing (1866‐1943), professor of pathology at Cornell, who when asked his thoughts about Broders’ concept of grading, said: “it is the practical application of the Hansemann principle.”^1^ Hearing this, Broders tracked down Hansemann's writings, hired translators, and then later acknowledged: “There is no doubt that what Dr Ewing said was true: that my work was the practical application of the Hansemann principle.”^1^ Hansemann, whose ideas were maligned during life, unfortunately died in 1920, almost certainly unaware that his work had such a practical application. Clearly, the importance of a facilitative environment cannot be over‐estimated, as the sad story of David von Hansemann demonstrates.

**Figure 2:**
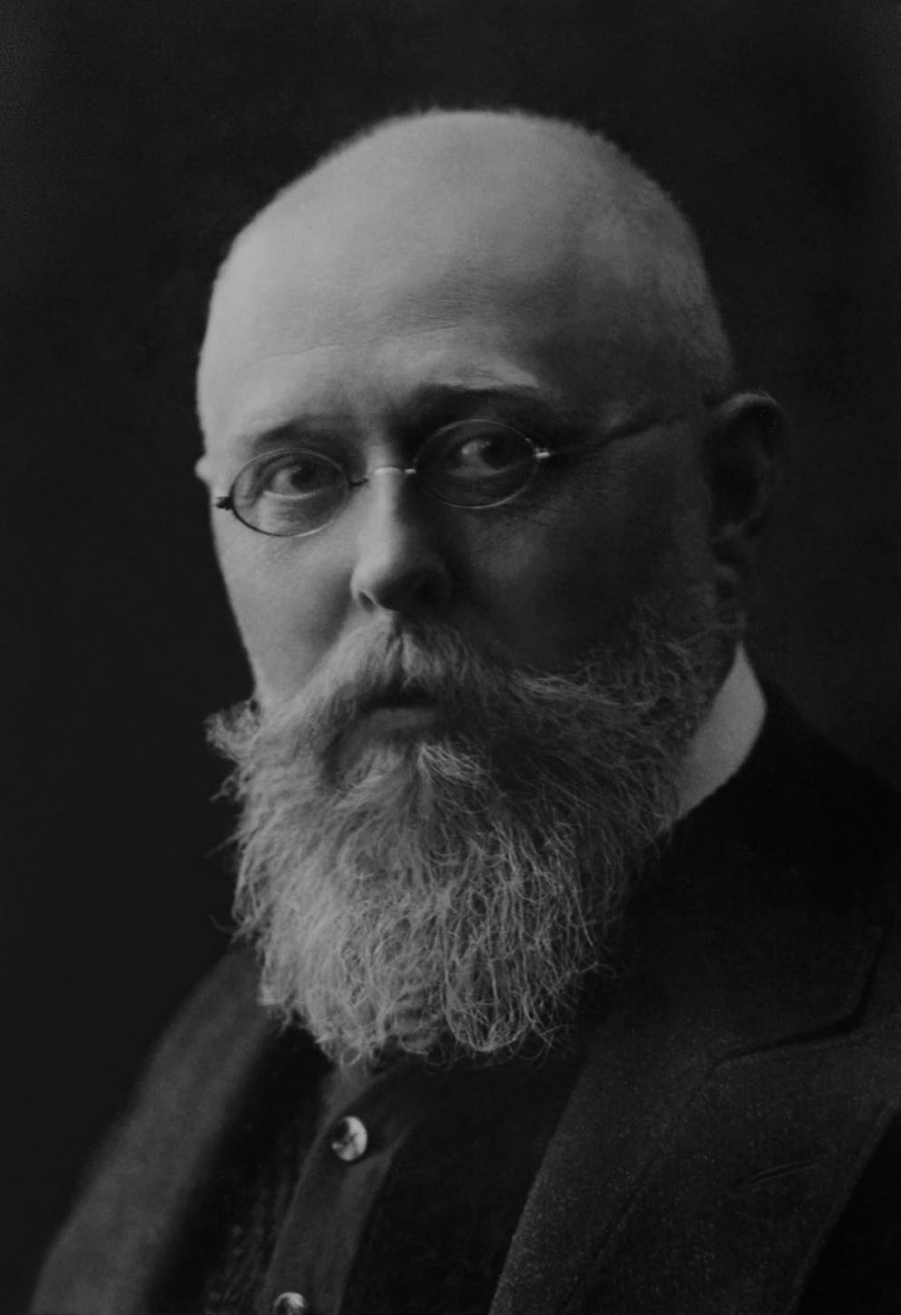
David Paul von Hansemann. Credit: von H. Noack, Berlin (photographer), photograph given to author by Prof. Dr Chr. Thierfelder at the Humboldt‐Universität zu Berlin in 1987; photograph predates 1923 and is in the public domain

In summary, a 100 years ago, Mayo Clinic pathologist Albert C. Broders published his first paper on tumor grading and initiated the long pathway toward personalized cancer care. Mayo Clinic laboratory director Louis B. Wilson, the ACS, and the ASCP facilitated its rapid and widespread implantation in hospitals across North America. The road toward personalized cancer care has enjoyed increasing traffic over the past century, but especially in the past couple of decades.

## Data Availability

Data sharing is not applicable to this article as it is a historical paper.
